# Comparison of microbial diversity and community structure in soils managed with organic and chemical fertilization strategies using amplicon sequencing of 16 s and ITS regions

**DOI:** 10.3389/fmicb.2024.1444903

**Published:** 2025-02-13

**Authors:** Ajay Kumar Mishra, Poonam Yadav, Sheetal Sharma, Piyush Maurya

**Affiliations:** International Rice Research Institute, South Asia Regional Centre, Varanasi, India

**Keywords:** metagenomics, soil sustainability, organic farming, amplicon sequencing, 16S-ITS

## Abstract

Soil microbial species diversity and distribution of microbial communities are vital for soil and crop health, nutrient cycling, availability, and subsequent plant growth. These soil dynamics are highly influenced and altered by various soil management practices, inputs, and agricultural techniques. In the present study, the effects of chemical and organic management practices on soil microbial diversity and community structure were examined and compared using amplicon sequencing of the 16S and ITS regions. Two contrasting soil samples were selected from each crop fields at the International Rice Research Institute-South Asia Regional Centre (IRRI-SARC) in Varanasi: one field followed conventional chemical fertilizer inputs, while the other implemented natural farming practices, including tillage, on-farm crop residue management, and water management. Soil samples from each field were analyzed for bacterial and fungal diversity. Our findings showed that the two differently managed soils exhibited distinct microbial community compositions, with the organically managed soil exhibiting a higher diversity of decomposer bacteria and fungi, showing 40 unique elements in organic soil samples and 19 in chemically managed soil. Natural farming practices also demonstrated a higher relative abundance of bacterial and fungal phyla. Our results emphasize the significance of sustainable soil management techniques, suggesting that organic inputs can increase soil microbial diversity and richness. The functional roles of these microbial communities in soil ecosystems and their potential impact on crop yield and nutrient cycling warrant further study.

## 1 Introduction

Soil is a complex and dynamic system essential for sustaining life on Earth. A critical component of soil, and a key indicator of its sustainability, is soil microbial communities. These communities play a vital role in soil sustainability (Hartmann and Six, 2023) and crop productivity by providing crucial ecosystem services (Bardgett and van der Putten, 2014), such as nutrient recycling, nutrient availability to plants (Poonam Srivastava et al., 2017), carbon sequestration, and disease control through direct and indirect mechanisms (Van Der Heijden et al., 2008; Sun and Badgley, 2019; Krause et al., 2023). Further, soil micro-biota contributes to plant stress tolerance, systemic resistance and immunity build-up (Singh et al., 2023). However, the composition and function of these microbial communities are significantly influenced by soil management practices, especially the type and rate of fertilizers applied (Hartman et al., 2008; Enebe and Babalola, 2020).

To restore the soil health and sustainability, many practices have been employed for soil management to promote sustainable agricultural development and enhanced yield (Montanarella, 2015). Traditional tillage methods widely practised in conventional farming practices disrupt soil structure, causing increased soil erosion and the imbalance of the global carbon cycle (Van Oost et al., 2007; Bednář and Šarapatka, 2018). Conservation tillage and fallow rotation systems have been introduced to improve soil organic carbon storage, enhance nutrient uptake, and increase crop yields (Hussain et al., 2021; Krause et al., 2022; Li et al., 2023). These practices also reduce carbon dioxide emissions, maintain soil ecosystem health, and play a critical role in mitigating climate change’s impact on agricultural production (Sun and Badgley, 2019; Wagg et al., 2014; Yadav et al., 2021).

Two widely used soil management approaches, organic and chemical fertilization, have contrasting effects on soil microbial diversity and structure. Organic fertilization, involving plant-or animal-derived materials such as compost or manure, adds organic matter and improves soil health (Lupatini et al., 2013a,b; Krause et al., 2023; Xu et al., 2022). It has been shown to enhance microbial diversity, increase enzyme activity, and improve soil physicochemical properties. For instance, in one study (Xu et al., 2022), organic cultivation increased the abundance of dominant bacterial phyla, such as Proteobacteria and Acidobacteria, as well as fungal phyla like Ascomycota and Basidiomycota. In contrast, chemical fertilization provides concentrated nutrients that can boost crop yields, but long-term use may lead to reduced microbial diversity, degraded soil structure, and increased greenhouse gas emissions (Singh et al., 2011; Liang et al., 2019; Sun and Badgley, 2019). Therefore, organic fertilization has a more sustainable impact on soil health than chemical fertilizers (Rousk et al., 2009).

Metagenomics has emerged as a reliable and powerful biotechnological tool to comprehensively evaluate the effects of these practices on soil microbial communities. It enables the study of microbial diversity by analyzing the genetic composition of entire microbial populations in soil ecosystems (Suyal et al., 2019). Advances in next-generation sequencing technologies, such as amplicon sequencing of the 16S and ITS regions, allow for rapid and detailed profiling of bacterial and fungal communities (Saharan et al., 2023). Metagenomics technique has been widely accepted to investigate how environmental factors, including chemical fertilization and organic soil management practices affect microbial communities (Rousk et al., 2009; Saharan et al., 2023).

The theoretical framework for this study integrates the Biodiversity-Ecosystem Function Theory (Loreau et al., 2002) and the Soil Health Paradigm (Doran and Zeiss, 2000), which highlight the role of microbial diversity in maintaining soil functionality and resilience. Adverse impact of climate change and increased pesticide use can severely disrupt microbial communities, reduce biodiversity and crop yield, and weaken soil health. It is highly evident now that climate change can alter temperature and soil moisture levels (Yadav et al., 2021), directly impacting microbial activity and diversity (Compant et al., 2010), while excessive pesticide use can lead to soil degradation and the loss of beneficial microbes (Saharan et al., 2023). So, it is highly needed to understand how various farming practices and soil amendments are influencing soil microbial diversity and how we can develop a sustainable agricultural system with these findings that can also withstand environmental stresses, maintain productivity, and reverse the negative impacts of pesticides and climate change on soil ecosystems. Additionally, microbial diversity and composition in agricultural soils vary with crop/plant species, existing environmental conditions, irrigation and soil amendments/inputs practices (Majumdar et al., 2024). Microbes, especially in the rhizosphere and endosphere, influence plant growth and stress resilience (Upadhyay et al., 2018). Studies supported that organic farming promotes higher microbial diversity, thus enhancing crop/plant health yield (Bulgarelli et al., 2012; Edwards et al., 2015; Saharan et al., 2023).

This study aims to investigate the differences in microbial biodiversity and community structure between soils managed with organic and chemical fertilization strategies. The study’s novelty is based on the location, soil, and practice-specific study of the rice-wheat cropping system. There are few studies to justify the findings, so it is also very important to map the significance of such practices in agriculture. We hypothesize that organically managed fields with zero tillage will exhibit richer microbial diversity than chemically treated soils. Data were collected to assess differences in tillage frequency, microbial community composition, and photosynthetic bacterial inoculation in organic and conventionally managed plots over the rice-growing season.

Next-generation amplicon sequencing of 16S and ITS regions was used to identify and comparatively evaluate the variation in soil microorganisms’ community structure and diversity between these management systems. As this study integrates multiple organic management practices, including reduced and zero tillage, bio-input fertilization (Azolla, blue-green algae, vermicompost, and vermi-wash), diverse sowing methods, and reduced water use, in contrast to conventional practices makes it novel. This is the first study to examine the collective effects of these practices on soil microbes, offering valuable insights into sustainable soil management.

Our findings will have important implications for developing sustainable soil management practices that prioritize promoting healthy microbial communities (Yang et al., 2014). The integration of practices and technologies can result in more accessible and better soil and agricultural production system management that is more sustainable.

## 2 Materials and methods

### 2.1 Site description and sample collection

Two experimental fields were selected for soil sample collection: an organically managed paddy field and one conventionally managed paddy field. Organic practice has been done at this location for the last 3 years. The study was conducted during the year 2020–21 and 2021–22 at the agri-experimental farm of the International Rice Research Institute South Asia Regional Centre, Varanasi (25.302877–82.947973, 83 m above mean sea level), Uttar Pradesh, India.

The min-max temperatures during the experimental year range between 15.5°C and 36°C (mean values), respectively. Annual rainfall for the same period was between 900 and 1,113 mm range, and most of the rains (>70%) occurred during the monsoon months of July to September. Soil samples were randomly collected from (upper plow layer 0–15 cm) each replicated field trials and plots, and processed for 16S and ITS metagenomics study. Tillage, seed rate, cropping system and agronomic management practices followed in organic and conventional farming systems and the five practices, treatment and crop establishment table and initial soil properties are provided in Tables 1–3, respectively. Treatment under the natural farming were combined as various tillage and crop residue management practices along with alternated wetting-drying irrigation practices whereas in conventional practices traditional irrigational knowledge and use of chemical pesticides were applied on the Rice-Wheat-Mung cropping systems for the assessment.

**Table 1:** Experimental design, and related agronomic management practices followed in organic and conventional farming systems.

Management practices	Scenario 1 (NF)*	Scenario 2 (CF)*
Field preparation	Rice: CT Wheat: RT Mung bean: ZT	Rice: CT Wheat: CT
Seed rate (kg ha^−1^)	Rice: 25 Wheat: 100 Mung bean: 20	Rice: 25 Wheat: 100 –
Sowing method	Manual transplanting in rice and seed drill sowing in wheat and mung bean	Manual transplanting rice and broadcasting of wheat
Crop geometry and spacing (cm)	(22.5–22.5–45)	Random
Fertilizer (NPK) in kg-ha^−1^	Nutrients applied through bio-input (Azolla, BGA, Vermicompost, Vermiwash)	Rice: 120:60:40
Water management (no. of Irrigation)	Rice: AWD (20–25 irrigations) Wheat: 3–4 Mung bean: 1–2	Rice: Soil was wet for up to 20 days after sowing irrigation applied at hair-line cracks (30–35 irrigations). Wheat: 3–4
Crop Varieties	Rice: Arize 6,444 Gold Wheat: PBW 187 Mung bean: Virat	Rice: Arize 6,444 Gold Wheat: PBW 187

*Scenario 1—Natural farming (NF refers to reduced tillage, crop diversification, Alternate wetting drying); CT, Conventional tillage; RT, Reduce tillage; ZT, Zero tillage practices; and AWD, Alternate wetting drying (It is a water-saving method in which irrigation water is applied after the water below 10 cm from the pipe has disappeared). Scenario 2—Conventional farming.

**Table 2:** Crop establishment method, cropping pattern and residue management under two different scenarios.

Scenarios with farming treatments	Crop rotation	Tillage	Crop establishment method	Residue management	Water management
Scenario 1 (NF)*	Rice-wheat-mung bean	Rice: CT Wheat: RT Mung bean: ZT	Rice: Puddled transplanted rice (PTR) with random geometry Wheat: Reduce tillage (RT) – line seedling Mung bean: Zero tillage (ZT) with row geometry	Rice: 30–40% retained Wheat: 10–20% anchored residue retained Mung bean: Full incorporated	AWD
Scenario 2 (CF)*	Rice-wheat-fallow	Rice: CT Wheat: CT	Rice: Puddled transplanted rice (PTR) with random geometry Wheat: Conventional till (CT) with broadcast seedling	All crop residue removed	Border irrigation

*Scenario 1—Natural farming (NF); CT, Conventional tillage; RT, Reduce tillage; ZT, Zero tillage practices; and AWD-Alternate wetting drying. Scenario 2—Conventional farming (CF).

**Table 3:** Initial soil properties of the agriculture field before crop establishment.

Soil properties	Method used	Before crop sowing		After crop harvest	
		0–5 cm	5–15 cm	0–5 cm	5–15 cm
Sand (%)	Particle size analysis	≈48		≈48	
Slit (%)		≈74		≈73	
Clay (%)		≈27		≈24	
Textural class	USDA triangle	Sandy clay loam		Sandy clay loam	
Bulk density (g/cm^3^)	Blake and Hartage	1.48 ± 0.03	1.49 ± 0.02	1.47 ± 0.02	1.49 ± 0.01
pH (1:2.5)	Glass electrode Ph meter	7.84 ± 0.03	7.84 ± 0.03	7.83 ± 0.02	7.84 ± 0.01
EC (uS/m)	Conductivity bridge	159.3 ± 1.07	164.9 ± 2.11	168.7 ± 2.11	174.9 ± 3.06
Soil organic carbon (SOC%)	Walkley and Black Method	0.42 ± 0.03	0.38 ± 0.02	0.46 ± 0.04	0.40 ± 0.03
Available P (kg/ha)	Olsen’s Method	66.2 ± 13.6	68.4 ± 11.2	71.3 ± 11.0	78.1 ± 15.6
Available N (kg/ha)	Kjeldahl Method	153.1 ± 2.66	156.2 ± 3.45	161.2 ± 6.01	166.0 ± 5.13
Available K (kg/ha)	Ammonium Acetate Methods	160.4 ± 18.0	163.2 ± 12.3	173.2 ± 15.8	181.2 ± 18.8

The collected soil samples were homogenized and spread out in trays to remove any extraneous materials, such as small stems, root fragments, and leaves. Afterward, the samples were sieved and stored in plastic bags for further analysis. The soil collection process, and analysis methods were standardized to ensure consistency for both the treatments plots. Following sample collection, soil properties such as pH, organic matter content, available phosphorus (P), and available potassium (K) were analyzed (Li et al., 2023).

During sample collection, 3 samples/sites/replicates were collected and pooled from organically fields and same was done for conventional fields. Initial soil parameter of agricultural soil before sowing and harvest were done (Table 3).

### 2.2 Comparison of organic soil microbial load with conventional field

Sampled soil was further assayed for microbial count using the standard serial dilution method (Saharan et al., 2023). A hundred milligrams of each soil sample were added to 900 mL of sterilized distilled water. After homogenization for 30 min, this solution was decimally diluted (10–1 to 10–9) and aliquots of the resulting solution, i.e., 10–9, were used for plating on appropriate culture media (nutrient agar) by pour plate method. After incubation at 30 ± 0.5°C, the colony-forming units (CFU) were counted.

### 2.3 DNA extraction, purification and amplification

DNA extraction, amplification and purification was conducted at Biokart India Pvt. Ltd. DNA was extracted from each treatments replicates using a commercial soil DNA extraction kit of Xploregen as per the manufacturer’s recommendations. To minimize DNA extraction bias, DNA samples from three replicates were pooled. Using Nano Drop and Gel electrophoresis, the DNA isolated from the samples was evaluated for quality. High-quality DNA was indicated by Nano Drop measurements (Absorbance at 260 nm/Absorbance at 280 nm) of about 1.8 to 2.0. The DNA samples were broken up into 600 bp length fragments using the KAPA fragmentation method (KAPA HyperPlus Kit including KAPA Frag Enzyme, KAPA Frag Buffer (10X) Conditioning Solution; a proprietary enzymatic systems) for library preparation by following the protocol mentioned in the Kit. Using a Hyper-Prep-plus ERAT (end-repair and A-tailing) enzyme combination, the fragmented materials were treated for end repair and A-tailing. For end-repair and A-tailing, 7 μL ERAT buffer and 3 μL ERAT enzyme mix was added to each sample (also previously fragmented gDNA samples) and incubated at 20°C for 30 min (end-repair) followed by 65°C for 30 min (A-tailing). Next, the adapters were introduced and ligated to the end repaired DNA segments using DNA ligase just after the end repair and A-tailing. Illumina primers were used to library amplify the adapter ligated samples. The V3V4 region of the 16S rRNA gene and the ITS region (In this study, we used the ITS region as a genetic marker for assessing fungal diversity in soil samples, following established methods for fungal community analysis) were amplified using universal primers (Caporaso et al., 2011; White et al., 1990). For bacterial diversity, the universal primers targeting the 16S rRNA gene (V3–V4 regions) were used: 341F (CCTACGGGNGGCWGCAG) and 805R (GACTACHVGGGTATCTAATCC). For fungal diversity, the Internal Transcribed Spacer (ITS) region was targeted using the universal primers: ITS1-F (CTTGGTCATTTAGAGGAAGTAA) and ITS2 (GCTGCGTTCTTCATCGATGC).

#### 2.3.1 ITS

To perform the amplification, 40 ng of the isolated DNA and 10 pM of each primer were used. Denaturation was started at 98°C, followed by four cycles of amplification (denaturation at 98°C for 15 s, annealing at 60°C for 30 s, extension at 72°C for 30 s), and finally final extension at 72°C for 1 min. For the detailed protocol and steps, Saharan et al. (2023) can be followed.

### 2.4 Illumina sequencing reads and taxonomic analysis

Two types of amplicon sequencing were performed (a) 16S rRNA gene metagenomics sequencing and (b) ITS metagenomics sequencing to understand the diversity of bacterial and fungal community in both kind of samples (organic and chemical). The amplicons from each sample were purified with Ampure beads (Beckman Coulter, Brea, CA, United States) to remove unused primers. An additional 8 cycles of PCR was performed using Illumina barcoded adapters (using primers complementary to the Illumina flow cell adapters) to prepare the sequencing libraries. For this, PCR products were purified using Ampure XP beads. The beads were added to the PCR products at a 1.8:1 ratio, followed by incubation to bind the DNA. The beads were separated using a magnetic stand, and the supernatant was discarded. After two ethanol washes (70%) and brief air drying, the DNA was eluted with nuclease-free water or buffer and collected for further use as per the protocol of Beckman Coulter (Zhang et al., 2020). Libraries were purified using Ampure beads and quantitated using Qubit dsDNA High Sensitivity assay kit (Thermo Fisher Scientific Inc., Waltham, Massachusetts, United States). Sequencing was performed using Illumina Miseq with 2 × 300 PE v3-v4 and ITS sequencing kit. The PCR products were sequenced using Illumina MiSeq platform (Illumina, San Diego, CA, United States) by following the standard protocol,^1^ and the resulting raw sequence reads of Sample 1 and Sample 2 were quality checked using FastQC (Version 0.11.9) and MultiQC (Version 1.10.1) tools. Adaptors from the raw data were trimmed out with Trimgalore version 0.6.6 and further were processed using Biokart Metagenomics pipelines to get the final OTU (Operational Taxonomic Units) tables. Taxonomy was assigned using the NCBI and UNITE reference databases for 16S and ITS sequences with the confidence of 0.05, respectively (Nilsson et al., 2019; Schloss et al., 2009).

For Illumina sequencing, primers were designed with specific overhangs to allow for indexing and adapter ligation during the sequencing workflow. The primers were prepared by incorporating Illumina adapter sequences into the forward and reverse primers used for amplifying the target regions of bacterial 16S rRNA and fungal ITS genes.

The forward primer contains the i5 overhang sequence (5′-TCGTCGGCAGCGTCAGATGTGTATAAGAGACAG-3′) and is combined with the specific region targeting sequence for either the bacterial 16S or fungal ITS region. Similarly, the reverse primer includes the i7 overhang sequence (5′-GTCTCGTGGGCTCGGAGATGTGTATAAGAGACAG-3′) followed by the target region-specific primer. These overhangs facilitate the addition of sequencing adapters during library preparation, which are essential for Illumina sequencing.

### 2.5 Calculation of rarefaction and diversity indices

Rarefaction curves are plots of the number of individuals on the x-axis (sequence count) against the number of species on the y-axis (species richness). Sample sizes (N) may differ, but the relevant sections of the curves can still be visually compared. Alpha diversity metrics, such as richness and diversity indices (Chao1, Shannon, Simpson and Fisher), were calculated for each sample (Deluz et al., 2020). Past4.03 was used to generate the plots related to the alpha diversity indices and rarefaction curve with default parameters.

### 2.6 Statistical and comparative analysis

Alpha diversity indices were visualized using box plots to illustrate the distribution of diversity across both farming practices. Statistical significance was determined with *p*-values, considering *p* < 0.05 as significant. Heatmap was generated to analyse the pattern of higher and lower abundance spread between the samples using Euclidean distance method with top 50 organisms at genus level of taxonomy with ClusVis.^2^ Comparative analysis was done to find the unique and common taxon between the samples in terms of 16S and ITS metagenomics. The analysis entirely depends on the category of organisms at genus level. The comparative Venn diagram was built using the tool Venny 2.1.^3^

## 3 Results

### 3.1 Soil parameters and physicochemical properties

Soil sample were collected from organically and conventionally farmed fields and subjected to soil nutrients analysis before crop sowing and after crop harvesting. On comparing the soil data from both fields, there were no any significant changes observed in sand, slit, clay, texture class, bulk density and pH before sowing and after harvesting and along the soil depth profile. Whereas, EC was increased at initial and final sampling along the soil depth. SOC was found slightly increased at two time points. Similar trend as SOC was followed by N-P-K concentration along with the soil depth profile (Table 3). Soil properties take a significantly longer time to change their natural characteristics, that is why in our case no considerable changes in soils were noted.

### 3.2 Taxonomic analysis

**Figure 1:**
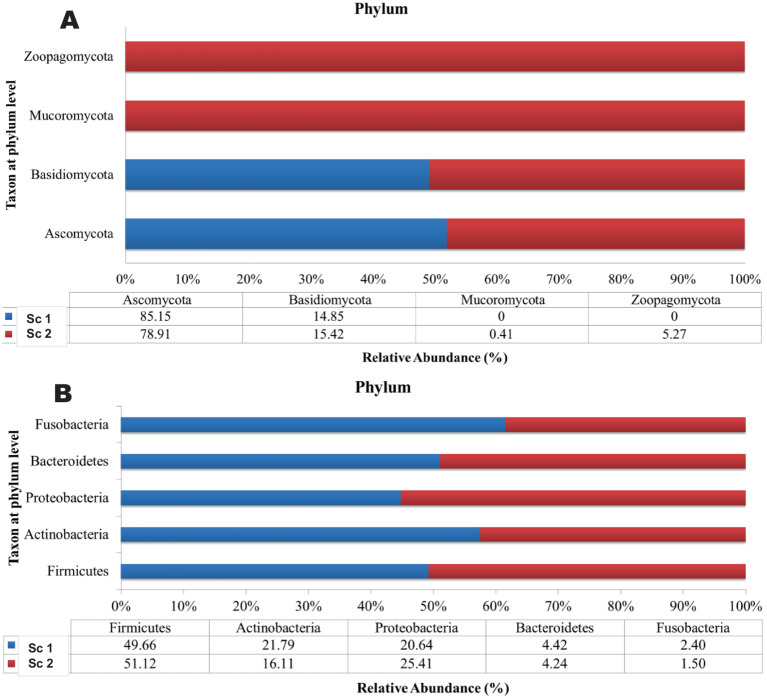
The plots reveals that the most abundant phylum of bacteria **(A)** is Firmicutes [Sc 1(49.7%) and Sc 2 (51.12%)]. The most abundant phylum of fungi **(B)** is Ascomycota [Sc 1(85.15%) and Sc 2 (78.9%)].

**Figure 2:**
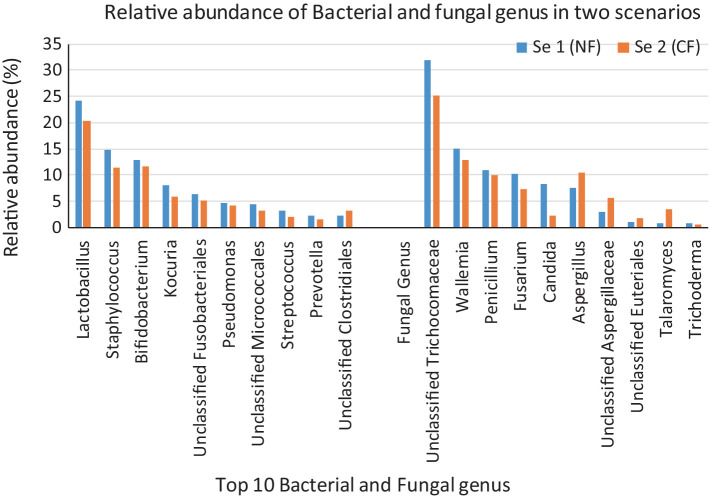
The above 100% stacked bar plots gives us the idea of the top 10 most abundant bacteria and fungus present in the Sc 1 and Sc 2. 16S study revealed that Lactobacillus is the most abundant bacteria observed in Sc 1(25.17%) and Sc 2 (20.45%). ITS study says that most abundant fungus observed in Sc 1 (31.92%) and Sc 2 (25.17%) is Unclassified Trichocomaceae.

A total of 200,171 bacterial sequences and 17,058 fungal sequences were obtained for each sample after quality filtering. The taxonomic profiling reveals a total of 724 OTUs bacteria and 230 OTUs of fungus were observed in amplicon based metagenomics study of soil sample from scenario 1 and 2. At the phylum level, both the organic sample (Sc 1) and the conventional sample (Sc 2) had a higher relative abundance of *Firmicutes (49.66; 51.12%)*, *Actinobacteria (21.79; 1,611%)* and *Proteobacteria (20.64; 25.41%)* bacteria (Figure 1) whereas in fungus category *Ascomycota* (85.15; 78.91%) and *Basidiomycota (14.85; 15.42%)* showed the highest relative abundance. If analysing the data at the genus level, both the organic sample (Sc 2) and the conventional sample (Sc 1) had a higher relative abundance of *Lactobacillus (24.17; 20.45%)* and *Pseudomonas (14.72; 11.31%)* (Figure 2). The ITS analysis showed that both the organic sample (Sc 2) and the conventional sample (Sc 2) had a higher relative abundance of Ascomycota and Basidiomycota (Figure 1). At the genus level, both the organic sample and the conventional sample had a higher relative abundance of Unclassified Trichocomaceae and *Aspergillus* (Figure 2).

### 3.3 Rarefaction curve

**Figure 3:**
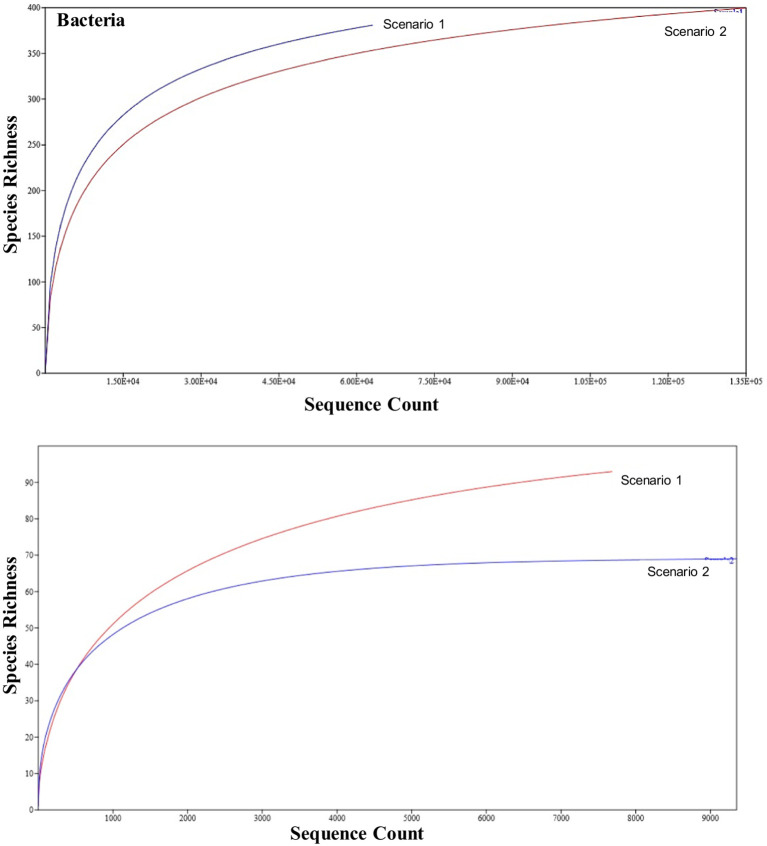
Representation of rarefaction curve for the NF (Sc 1) and conventional sample (Sc 2) on the basis of bacterial and fungal results using Past4.03.

The number of individuals on the x-axis (sequence count) was plotted against the number of species on the y-axis (species richness) to visualize the rarefaction curve of the organic and conventional samples. Sample sizes (N) may differ, but the relevant sections of the curves can still be visually compared. In terms of 16S study the Sc 1 has higher sequence count and higher species richness compared to Sc 2 in terms of 16S metagenomics study whereas in ITS study the Sc 1 has higher species richness and lower sequence count while Sc 2 has higher sequence count and lower species richness (Figure 3).

### 3.4 Diversity analysis

The analysis revealed that the conventional sample (Sc 2) had higher microbial diversity and richness than the organic sample (Sc 1) (Table 4), as indicated by the Simpson, Shannon, Fisher and Chao1 indices (Chao and Lee, 1992; Fierer et al., 2005). Alpha diversity is used to measure the diversity present within a sample or community. Alpha diversity can be characterized via the total number of species (richness), the abundances of the species (evenness) or measures that considered both richness and evenness. How these measures estimate the diversity is need to be considered when performing alpha-diversity analysis. Chao1 estimate the richness by inferring out the number of rare organisms that may have lost due to under sampling. The indices such as Shannon, Simpson and Fisher infer the number (richness) along with the abundance of organisms (evenness) and measured to describe the actual diversity of a community (Pielou, 1966).

**Table 4:** Representing the alpha diversity measure of organic and conventional soil samples for both bacterial and fungal communities.

Alpha diversity index	Bacteria		Fungi	
	Sc 1	Sc2	Sc 1	Sc 2
Simpson_1-D	0.9134	0.8856	0.8839	0.8386
Shannon_H	3.185	2.884	2.66	2.376
Fisher_alpha	53.94	50.66	10.1	14.88
Chao-1	435.6	433.6	70	101

Where: Scenario (Sc) 1: Natural farming; Sc 2: Conventional farming.

**Figure 4:**
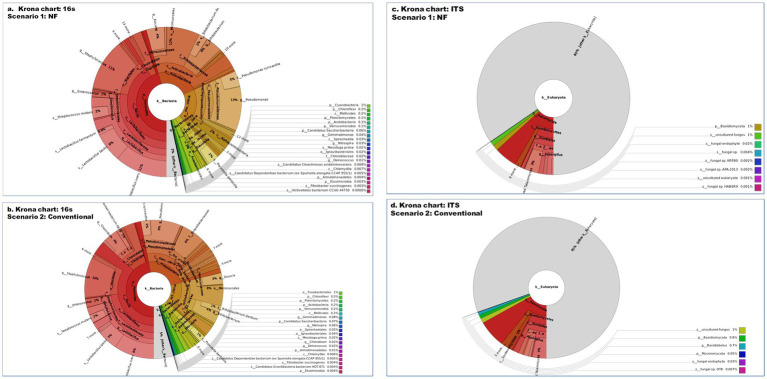
The phylogenetic tree diagram of soil bacterial **(A,B)** and fungal **(C,D)** communities in organic farming (NF, Sc 1) and conventional farming (CF, Sc 2) as identified in 16S and ITS. The phylogenetic tree shows the overall sample, from the phylum to the genus (from the inner to the outer ring arranged in sequence). The size of the point represents the average relative abundance of the taxon, and the relative abundance of the first 20 taxa is also identified with letters.

**Figure 5:**
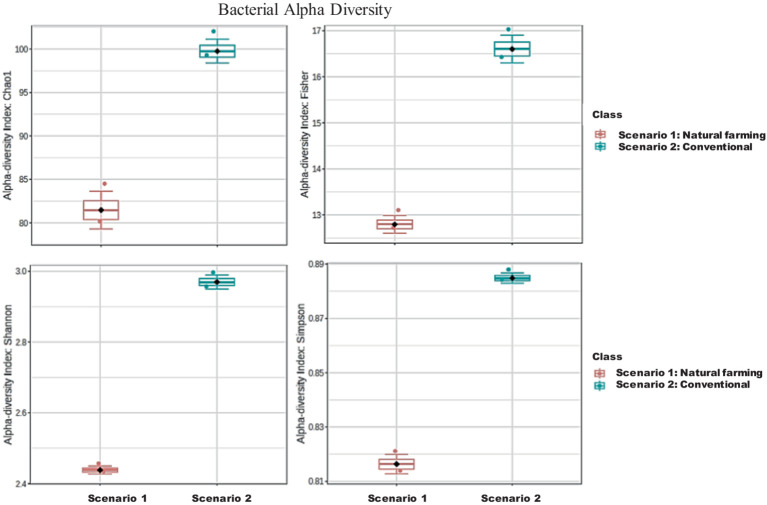
Alpha diversity of bacterial community in between NF (Sc 1) and conventional (Sc 2) soil samples.

**Figure 6:**
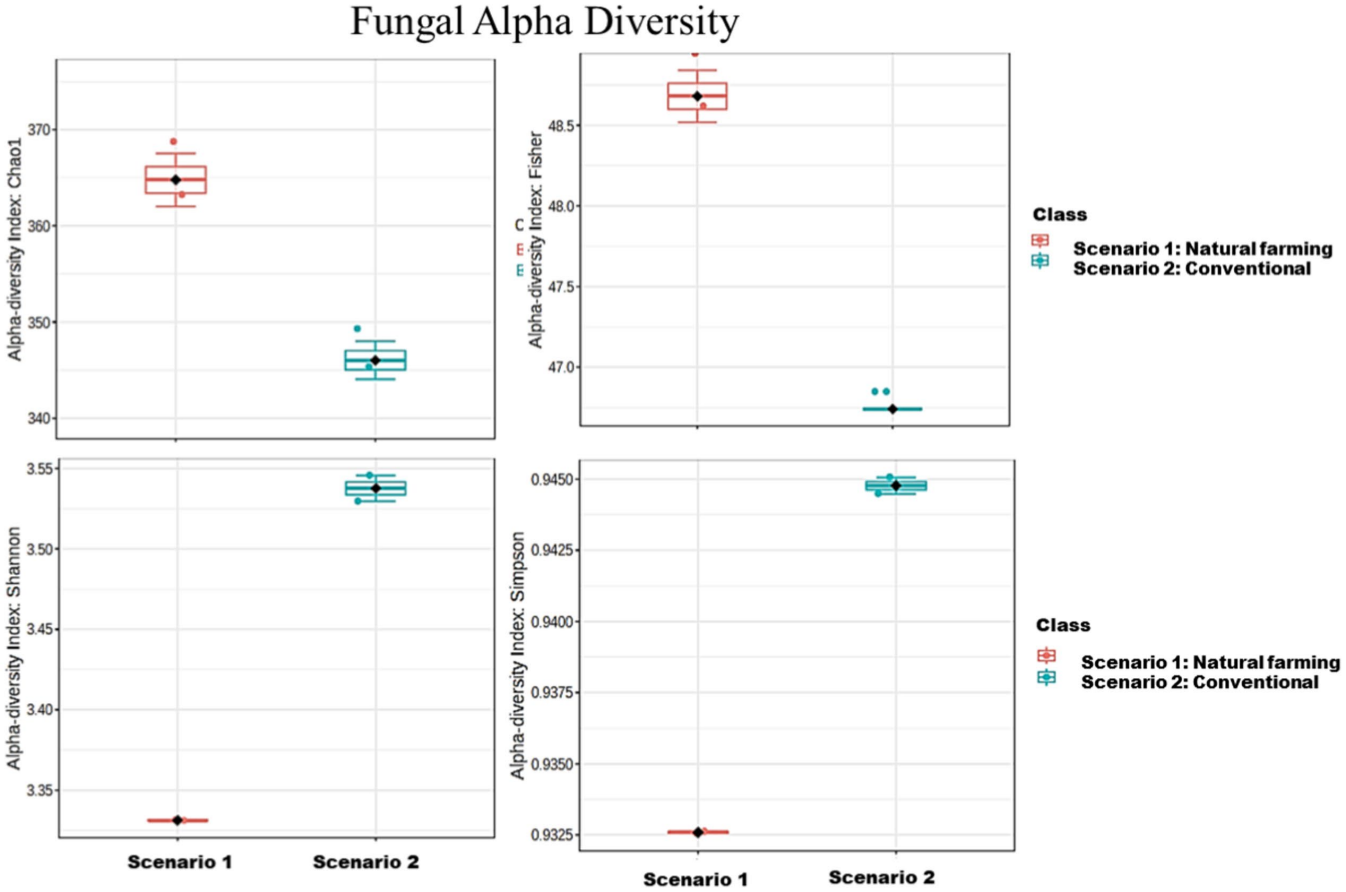
Comparison of Alpha diversity of fungal community between natural farming (NF) (Sc 1) and conventional (Sc 2) soil samples.

In terms of 16S metagenomics, even though the Sc 1 has higher sequence count and high species richness, but from the alpha diversity we can observe that Sc 2 has higher diversity. While in terms of ITS metagenomics Sc 1 has higher diversity compared to Sc 2 and, Sc 2 has higher abundance (Figures 4–6).

### 3.5 Comparative studies

Top 50 soil micro-organisms were clustered using correlation distance and average linkage with unit variance scaling applied on to the sequence abundance to construct the heatmaps. The heat map infers,

1. *Differences in bacterial and fungal community composition:* The heatmap reveals the differences in the abundance of different taxa between the organic and conventional soil samples. Among the top 50 organisms there are 44 taxa of bacteria which are more abundant in organic soil sample and there are 34 taxa of fungi which are more abundant in organic sample compare to the conventional soil sample (Sc 2), this could indicate that the farming practices used in conventional agriculture are affecting the composition of the soil microbiome.
2. *Relationships between different taxa:* This heatmap also reveals the patterns of co-occurrence or exclusion between different bacterial and fungal taxa. There are 44 bacterial taxa and 34 fungal taxa that are consistently found to be abundant in Sc 01, this could suggest that they have a mutualistic relationship and the same applies for Sc 02

**Figure 7:**
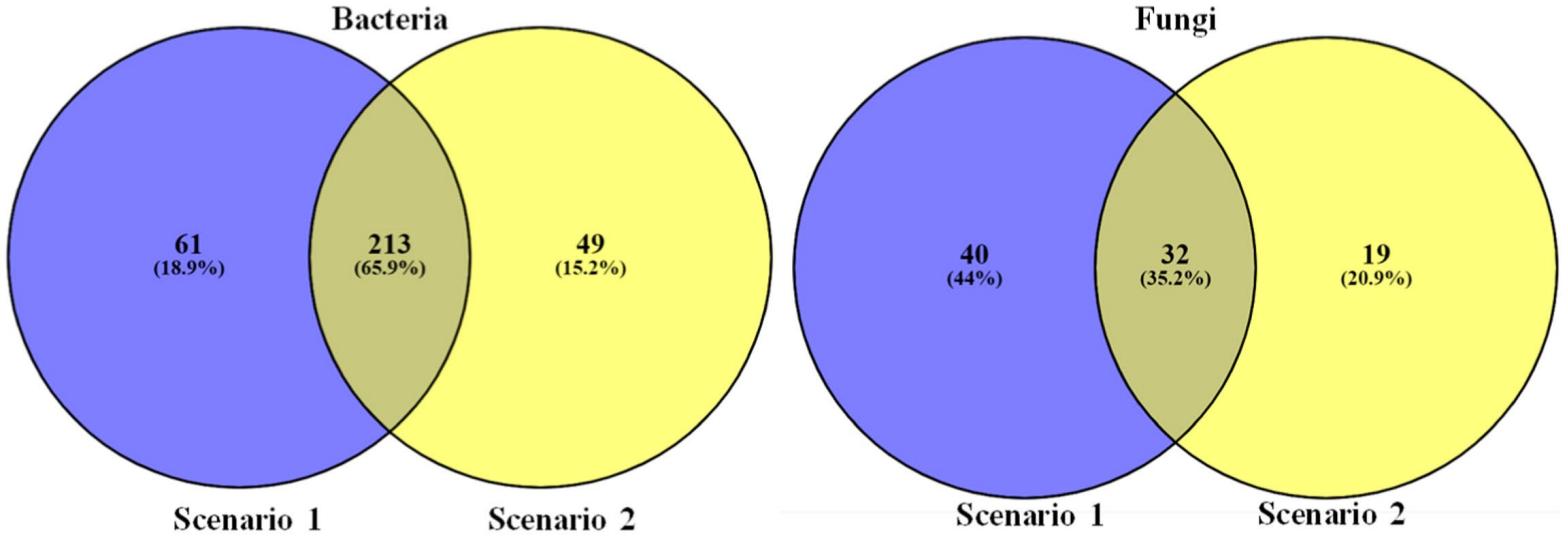
Venn diagrams representing the comparison of unique and common taxa between the two farming scenarios.

Samples were then compared on the basis of higher abundance. Taxon with 0 abundance are eliminated to find which among the organism are unique to each sample of study and observation (see Figure 7).

### 3.6 Crop yield

On comparing the two contarsting crop productions systems and practices, it was observed that chemical input based agriculture practises in the CF system showed more yield in comparison to the nature based inputs and management practices in NF scenarios. Our data observation and analysis for the year 2020–21 and 2021–22 shows that organic amendments increased the crop yields for the 2021–22 as compared to 2020–21, specially for rice crop approx (Table 5). 17% increase was noted. Wheat (3.12 to 3.45 t/ha) and Mungbean (0.30 to 1.71 t/ha) were also observed with an increase in crop yield though increase in yield of wheat was not significant. Rice equivalent was also increased in NF from 2020–21 to 2021–22 (7.33 to 8.64 t/ha). Whereas, there was a significant difference in rice equivalent system yield under the CF and NF for both the experimental years. It is a well-known fact that crop is drop in crop yield under the organic practices but here as mentioned in the treatment table, multiple approaches were combined (Tables 1, 2) and tested that gave enhanced yield in next year. This may be because of the established soil sustainability and health under the NF practices. Modern agricultural is going through lots of challenges and transforming our food system for the sustainable production is crucial. Also, the food production systems should produce the appropriate quality and quantity without challenging the environmental resources. Additionally, focus of sustainable agriculture practices is to have low Global Warming Potential (GWP) and GHG emission factors (Sun and Badgley, 2019). In this regard, many organic/natural and circular bio-economy based agriculture are proved to be climate friendly. A reduced yield is compensated by the low input and management cost. To this, an increased crop yield can be recorded after 3–4 years of natural and organic practices.

**Table 5:** Crop yield under different scenarios in the year 2020–22 (Unit: t/ha).

Crop	2020–21		2021–22	
	(CF)^*^	(NF)^*^	(CF)^*^	(NF)^*^
Rice (Arize 6,444 gold)	5.97 ± 0.36^a^	2.98 ± 0.18^b^	5.88 ± 0.29 ^a^	3.48 ± 0.16 ^b^
Wheat (DBW 187)	4.12 ± 0.20^a^	3.12 ± 0.16^b^	4.41 ± 0.18 ^a^	3.45 ± 0.17 ^b^
Mungbean (Virat)	–	0.30 ± 0.02^a^	–	1.71 ± 0.03 ^a^
Rice equivalent system yield^**^ (t/ha)	10.3 ± 0.57^a^	7.33 ± 0.42^b^	10.3 ± 0.46 ^a^	8.64 ± 0.37 ^b^

^*^CT, Conventional farming; NF, Natural farming (Unit: t/ha); Where: Scenario (Sc) 1: Natural farming; Sc 2: Conventional farming. Different alphabetical symbols against the data are denoting the significant difference between the data.

**Rice equivalent system yield is a practice to compare the yield of different crops by converting the yield of non-rice crops into an equivalent rice yield.

### 3.7 Soil microbes-related parameters

Soil properties related to the microbial action has also been reported to improve under the Sc 1 as compared to the Sc 2 (Table 6). Soil microbial biomass carbon and nitrogen (SMBC and SMBN) along with dehydrogenase, alkaline phosphatase, urease and TGRSP were reported in increased amount (73.51; 7,431; 36.81; 13.43 ± 4.54; 185.55 ± 1.5; and 2.23 ± 0.16, respectively) in the naturally managed soil as compared to the conventional soil (40.85 ± 5.14; 34.18 ± 16.11; 25.85 ± 0.88; 7.96 ± 0.62; 180.55 ± 2.58 and 1.78 ± 0.09, respectively). Soil enzymes are the indicators of the soil sustainability, fertility and health and organically soil shows higher activities of the microbial community under the degradable materials. While chemically hazardous inputs and intensively managed farming systems can greatly reduce the microbial population and biomass, application of organic amendments (crop-residue) and manure is a potential strategy to strengthen the microbial biomass and improve soil health and resilience (see Table 7).

**Table 6:** Effect of cropping system and different agriculture practices on soil microbial properties after 1 year of experiment.

Treatments	SMBC (μg^−1^ g^−1^)	SMBN (μg g^−1^)	Dehydrogenase μg TPF h^−1^ g^−1^	Alkaline phosphatase μg p-g^−1^ soil h^−1^	Urease mg urea g^−1^ soil h^−1^	Total glomalin related soil protein (TGRSP)
Sc 1 (NF)	73.51 ± 23.3	74.31 ± 3.54	36.81 ± 0.37	13.43 ± 4.54	185.55 ± 1.5	2.23 ± 0.16
Sc 2 (CF)	40.85 ± 5.14	34.18 ± 16.11	25.85 ± 0.88	7.96 ± 0.62	180.55 ± 2.58	1.78 ± 0.09

Scenario (Sc) 1: Natural farming; Sc 2: Conventional farming.

**Table 7:** List of top 10 genus of bacteria and fungi with their relative abundance (%).

Sl. No.	Top 10 bacteria (genus)	Sc 1 (NF)	Sc 2 (CF)	Top 10 fungi (genus)	Sc 1 (NF)	Sc 2 (CF)
1	*Lactobacillus*	24.17	20.45	Unclassified *Trichocomaceae*	31.92	25.17
2	*Pseudomonas*	14.72	11.31	*Aspergillus*	15.12	12.82
3	*Staphylococcus*	12.89	11.57	*Wallemia*	11.02	10.03
4	Unclassified *Micrococcales*	8.12	5.89	Unclassified *Aspergillaceae*	10.29	7.34
5	*Bifidobacterium*	6.43	5.06	*Penicillium*	8.17	2.28
6	*Streptococcus*	4.75	4.08	Unclassified *Eurotiales*	7.55	10.55
7	*Kocuria*	4.52	3.20	*Fusarium*	2.95	5.66
8	*Prevotella*	3.19	2.11	*Talaromyces*	1.10	1.70
9	Unclassified *Fusobacteriales*	2.32	1.44	*Candida*	0.84	3.54
10	Unclassified *Clostridiales*	2.24	3.21	*Trichoderma*	0.82	0.54

## 4 Discussion

Various activities, such as agricultural practices, significantly alter soil microbial function and communities, causing heavy use of toxic chemicals in crop fields and environmental pollution (Teng and Chen, 2019). The difference in the taxonomic profiles between the organic and conventional soil samples, as determined by 16S and ITS metagenomics, may be attributed to differences in the management practices between the two soil types. *Firmicutes* and *Actinobacteria* are commonly associated with soils that are high in organic matter, and the increased levels of these phyla in the organic soil sample may be indicative of the presence of more decomposing organic material, which could contribute to soil fertility (Fierer and Jackson, 2006). On the other hand, *Ascomycota* and *Basidiomycota* are fungal phyla commonly found in soils and are involved in the decomposition of organic matter (Xu et al., 2022). These phyla may also contribute to the nutrient cycling processes in the soil, and their increased abundance in the ITS metagenomics data from the organic soil sample may indicate a higher level of organic matter decomposition in this soil type (Rousk et al., 2010).

The conventional soil sample, on the other hand, may have lower levels of organic matter and thus lower levels of *Firmicutes*, *Actinobacteria*, *Ascomycota*, and *Basidiomycota* (Xu et al., 2022). It is also possible that the conventional soil sample has been subjected to higher levels of disturbance or chemical inputs, which may have impacted the microbial community composition and diversity (Zhou et al., 2012). A larger number of microorganisms in organically treated soils (fungi and bacteria) and associated biomass carbon have been reported (Saharan et al., 2023). Meanwhile, soil data from chemical fertilizers have shown more effects on microbial composition and diversity.

In a contrasting farming practice experimental study, Enebe and Babalola (2020) reported the response of various maize-related microflora (bacterial, fungi and archaeal communities) to compost and inorganic fertilization as we analyzed. The study also reports that both fertilizer practices influenced the maize rhizosphere microbial community, but the organic amendments provided the most stable microbial community. Similar results were documented by Zhan et al. (2018), stating that a higher level of NPK input in the soil negatively affects the microbial community’s abundance and structure. Moisture and mineral content also play a significant role in decomposing bacteria diversity.

Overall, the differences in microbial community composition between the organic and conventional soil samples suggest that management practices can significantly impact soil microbiota. The higher abundance of *Firmicutes* and *Actinobacteria* in the organic soil sample and the higher abundance of *Ascomycota* and *Basidiomycota* in the ITS metagenomics data may be indicative of a more diverse and active microbial community in the organic soil, which could contribute to improved soil health and fertility. Other studies have also reported a taxonomically diverse group of decomposer soil microbes that are related to the *Firmicutes, Actinobacteria, Proteobacteria* and *Bacteroidetes* phyla (Zhan et al., 2018; Bernard et al., 2007; España et al., 2011; Semenov et al., 2012). Growth of *Proteobacteria* and *Acidobacteriota* was associated with the amount of/and increasing soil organic carbon content and limitation of nutrients in the system. Plant species-dependent effects were also noted to impact the soil fungal community significantly (Hannula et al., 2019; He et al., 2022). On the other hand, soil bacteria are found to show higher species richness than fungal communities in the same soil-quality habitat. It can be concluded that the bacteria are better adapted to non-extreme soil disturbances due to greater diversity (He et al., 2017).

**Figure 8:**
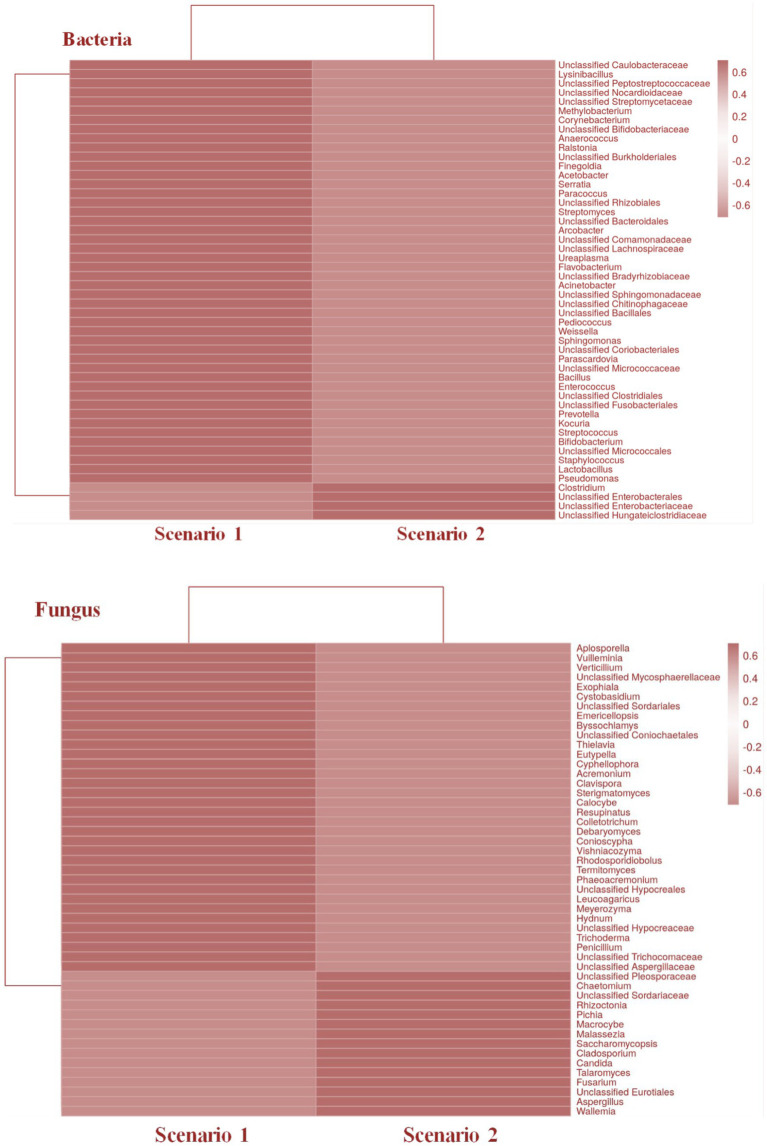
Heatmaps representing the taxonomical relationship between the two farming scenarios in both bacterial and fungal community.

In addition to fungi like *Aspergillus*, *Penicillium*, and *Fusarium*, the study by Saharan et al. (2023) also highlights beneficial bacteria such as *Bacillus* and *Pseudomonas* as critical contributors to soil health in zero-budget natural farming. This microbial diversity enhances nutrient cycling and plant resilience to stress. Previous studies by Singh et al. (2011) and Edwards et al. (2015) also support the beneficial roles of these bacteria in crop health and soil fertility, as they are also reported in our study (Figure 8).

Additionally, crop yield has been found to reduce, but on comparing the economics, natural practices are cost-effective. The findings demonstrate that soil microbial activity, represented by SMBC, SMBN, and enzyme activities (e.g., dehydrogenase, alkaline phosphatase, and urease), was significantly higher in naturally managed soils (Scenario 1) compared to conventional systems (Scenario 2). This suggests enhanced soil health, fertility, and sustainability under organic and water-saving (AWD) management practices (Majumdar et al., 2021, 2024; Das et al., 2024; Gunapala and Scow, 1998). Organic amendments like crop residues and manure promote microbial biomass, while intensive chemical inputs reduce microbial diversity and soil quality (Mandal et al., 2023).

Many people agree that organic farming is a successful approach to sustainably managing agriculture. Organic farming is typically thought to enhance crop/grain and soil quality. However, it is observed that farmers (special small land holding farmers) are hesitated to promote organic farming at large-scale because of the lower crop yields compared to conventional farming (based on the personal observations and interaction with the farmers). Though, yield cannot be the only deciding parameter to promote any practice, overall economic analysis and environmental sustainability along with farmers’ benefit should also be taken care in promoting them at larger level. The global adoption of environmentally friendly techniques will help preserve ecosystems and reduce the negative impacts of agrochemicals. However, further research must provide substantial evidence supporting these natural approaches for broader acceptance and implementation.

## 5 Conclusion

The qualitative and quantitative nature of the metagenomic DNA representing different environmental samples decides the success of metagenomic approaches. From the study, it can be concluded that use of different fertilizer use strategies in soil management systems has a significant role on the diversity and distribution of microbial communities. It can be a potential application-based practice in agriculture for soil health management under climate change impact management. The analysis of both 16S and internal transcribed spacer (ITS) amplicon sequencing data revealed distinct patterns of microbial community composition between the organic and chemical-based fertilizer treatments, with a greater diversity of bacteria and fungi observed in the soil treated with organic fertilizer. Results of the study provides valuable insight into the organically amended soil, and impact of different chemical based fertilization strategies on soil microbial communities and underscores the importance of sustainable soil management practices that prioritize organic fertilization. Further, more studies are needed to explore and support the functional implications of microbial communities in soil ecosystems and their potential for influencing crop productivity, nutrient cycling and fighting the climate change impacts.

## Data Availability

The original contributions presented in the study are included in the article/supplementary material, further inquiries can be directed to the corresponding author.

## Footnotes

^1^ http://www.illumina.com

^2^ https://biit.cs.ut.ee/clustvis/

^3^ https://bioinfogp.cnb.csic.es/tools/venny/
